# Preliminary Molecular Identification of a Predatory Bug, *Orius albidipennis*, Collected from Ornamental Plants

**DOI:** 10.1673/031.013.1101

**Published:** 2013-02-02

**Authors:** Samy M. Sayed, Metwally M. Montaser, G. Elsayed, Sayed A.M. Amer

**Affiliations:** 1 Faculty of Science, Taif University, Kingdom of Saudi Arabia; 2 Economic Entomology and Pesticides Department, Faculty of Agriculture, Cairo University, Egypt; 3 Zoology Department, Faculty of Science, Al-Azhar University, Egypt; 4 Zoology Department, Faculty of Science, Cairo University, Egypt

**Keywords:** biological control, ITSI, molecular identification, ribosomal DNA

## Abstract

*Orius albidipennis* (Reuter) (Hemiptera: Anthocoridae) is a generalist predator used for biological control of insects attacking ornamental plants. Molecular identification of this species using internal transcribed spacer 1 (ITS1) of ribosomal DNA was conducted for the first time. The complete sequence of ITS1 and fragments of its flanking 18S and 5.8S rDNA genes are reported herein. The estimated length of ITS1 of *O. albidipennis* was 305 bp. This spacer was nearly identical to its counterpart of *Orius* sp-Taif strain in spite of the difference in their length. The phylogentic relationships were determined using the maximum-likelihood method supported with strong bootstrap probabilities clustering of both taxa together. Further molecular markers could be useful to identify the Taif strain and support its sister relationship to the Egyptian *O. albidipennis*.

## Introduction

The anthocorid bugs (Anthocoridae: Hemiptera), sometimes referred to as flower bugs or minute pirate bugs, contain approximately 400–600 species, which are distributed worldwide (Schuh and Ŝtys 1991). Most species of Anthocoridae are predaceous as nymphs and adults (Anderson 1962; Lattin and Stanton 1992). Some species such as *Orius albidipennis* (Reuter) (Hemiptera: Anthocoridae), *O. laevigatus, O. strigicollis*, and *O. insidiosus* are used as biological control agents and are commercially produced and traded (Yasunaga 1997; Kim et al. 2008).

Some *Orius* species are sold commercially for augmentive biological control releases. *Orius* have been used successfully as a biological control in a number of greenhouse crops and open fields including sweet pepper, cucumber, and some ornamentals (Van de Veire and Degheele 1992; Chambers et al. 1993; Lorenzana et al. 2010). Accordingly, it is very important to evaluate this generalist predator systematically for managing its pest control.

Narita and Ohno (2000) identified adult females of the genus *Orius* independent of cuticular hydrocarbons by gas chromatography-mass spectrometry The molecular diversity and systematics of *Orius* are poorly understood. Honda et al. (1998) used the internal transcribed spacer 1 (ITS1) and its flanking rDNA genes to infer the relationship among 10 *Orius* species. Hinomoto et al. (2004) developed multiplex polymerase chain reaction (PCR) methods to identify five *Orius* species that occur commonly in Japan. They amplified ITS1 of the nuclear ribosomal DNA. Jung et al. (2010) studied the family Anthocoridae, including 9 species of *Orius*, by using molecular markers. Other investigations also used molecular markers to examine the the anthocorid family (Muraji et al. 2000a, b; 2004).

Sayed and Montaser (2012) used the ITS1 to identify an *Orius* species inhabiting west Saudi Arabia, but they could not identify which species it was. The authors however, named this taxon *Orius* sp-Taif Strain. To the best of our knowledge, there are no molecular systematic studies of *O. albidipennis* so far. This work, therefore, aimed to sequence ITS1 and its flanking rDNA genes for the Egyptian *O. albidipennis* and compare it to its counterparts of *Orius* sp-Taif strain, as well as other sequenced species, in order to construct a preliminary identification of *O. albedipennis* and relate it to the Taif strain.

## Materials and Methods

A well-identified and recorded specimen was taken from the laboratory of predators mass production, Faculty of Agriculture, Cairo University. The insect was reared as a commercial product and was morphologically identified as *O. albidipennis*. It was originally collected from a field in Cairo, Egypt. An individual *O. albidipennis* was dissected, the gut was removed and discarded, and the remainder was ground in 100 µl lysis buffer (5% Chelex-100 and 4µl of proteinase K (20µg/µl) in a 1.5-mL tube. The tube was incubated for 6 hours at 56° C followed by 10 minutes at 95° C, then centrifuged at 12000 rpm for 10 minutes (Muraji et al. 2004). DNA in the supernatant was spectrophotometrically measured (at 260/280nm), then used for PCR.

The resultant solution was electrophoresed on a 1.5% agarose gel in TAE (40mM Tris, 40mM acetic acid and 1mM ethylenediaminetetra acetic acid) and the gel was stained with ethidium bromide. 100 bp DNA Ladder (Biolabs) was used as a marker for the molecular weight size. The PCR product was then purified from gel with the use of a spin column according to the kit manual.

Sequencing reactions were performed in a MJ Research PTC-225 Peltier Thermal Cycler using a ABI PRISM. BigDyeTM Terminator Cycle Sequencing Kits with AmpliTaq-DNA polymerase (FS enzyme) (Applied Biosystems, http://www.appliedbiosystems.com/) following the protocols supplied by the manufacturer were used. A single-pass sequencing was performed on each template using the last mentioned PCR-primers. The fluorescent-labeled fragment was purified from the unincorporated terminators with an ethanol precipitation protocol. The sample was resuspended in distilled water and subjected to electrophoresis in an ABI 3730xl sequencer (Applied Biosystems). The obtained sequence was analyzed with the DNASIS-Mac program version 3.5 (Hitachi, http://www.hitachi.com/). After the sequence was obtained, its feature was identified by both Sequencher version 4 and MacClade version 4.08 programs (Sinauer Associates, Inc., http://www.sinauer.com/).

**Figure 1.**
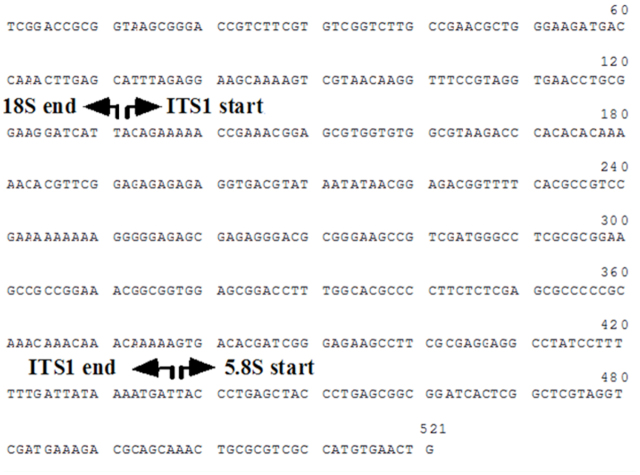
Features of the sequenced fragment of 18S rDNA gene, ITSI, and 5.8S rDNA gene from *Orius albidipennis*. High quality figures are available online.

Sequences of ITS1 and partial 18S rRNA gene for other *Orius* species were obtained from GenBank with their accession numbers as follow: *O. tristicolor* GU214727, *O. sauteri* AF061371, *O. nagaii* AF061369, *O. laevigatus* AF061366, *O. strigicollis* AF061372, *O. niger* AF061370, *O. majusculus* AF061367, *O. tantillus* AF061373, *O. pumilio* GU214728, *O. minutus* AF061368, *O.* sp-Taif strain HQ699724, and *O. insidiosus* GU214726. *Wollastoniella rotunda* (AF061375) was used as an outgroup to root the constructed relationship. The obtained sequences were aligned separately and manually using MacClade version 4. The major portion of ITS1 was unalignable among the different taxa, and only 43 bp were alignable and used in the analysis besides 131 bp from 18S gene. The tree analysis was done by the maximum-likelihood method in PAUP* 4.0b10 (Swofford 2002). In this analysis, heuristic searches with the TBR branch swapping and 10 random taxon additions were adjusted. The bootstrapping replicates were set to 1000 with simple additions.

**Figure 2.**
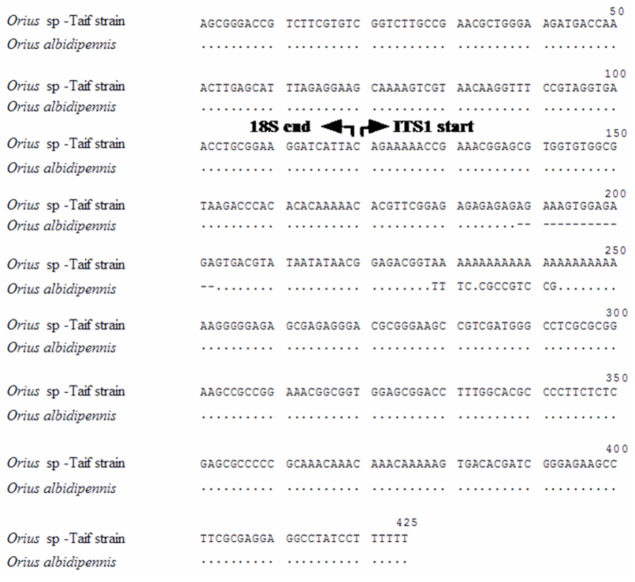
Sequence alignment of ITS1 and its flanking portion of 18S rDNA from *Orius albidipennis* and Taif strain. Dots indicate identity to the above sequence while dash denotes deletion. High quality figures are available online.

## Results and Discussion

Using the primers of Hinomoto et al. (2004), *O. albidipennis* gave a PCR product that showed similar size to its counterpart of some *Orius* species (data not shown). Sequencing of the PCR product showed that the amplified fragment acquired the typical arrangement of all *Orius* species sequenced so far. The first 131 bp were located in 3′ end of 18S rRNA gene, followed by 306 bp for the complete ITS1 and 74 bp for the 5′ end of 5.8S rRNA gene (Figure 1). The nucleotide sequence data reported in this paper will appear in the DDBJ/EMBL/GenBank nucleotide sequence databases with the accession number AB624550.

After the sequence of the present study was obtained, individual features were identified in light of their counterparts from other *Orius* species. As for identification of ITS1, the conserved sequences of both 18S and 5.8S rDNA genes that are typically located on either boundaries of the spacer were searched. The start of ITS1 was identified by searching for 5′- ACCTGCGGAAGGATC ATT-3′, which was found at the 3′-end of 18S rDNA gene as shown in Figure 1. For the 3′-end of the spacer, the sequence 5′TACCCTGAGCGGCGG-3′ was located at the 5′-end of 5.8S rDNA gene.

The ITS1 was unalignable among different *Orius* species and showed identity only between *O. albidipennis* and *O.* sp-Taif strain. The length of this spacer was 324 bp in the Taif strain and 306 bp in the Egyptian strain (Figure 1). The alignment of this spacer is, therefore, shown only for *O. albidipennis* and *O.* sp-Taif strain in Figure 2, with nearly complete similarity between the two taxa. The spacer showed 14 bases deletion at the position 70 and different substitutions at the sequence 5′-TTTCACGCCGTCCG-3′ in *O. albidipennis*.

ITS1 showed variable lengths among the different *Orius* species and it exhibited great ambiguity in its features; therefore, it was unalignable. Several investigations revealed variable lengths for ITS1 in the different species of the genus (Honda et al. 1998; Hinomoto et al. 2004; Muraji et al. 2004). The authors found that ITSl was 659 bp in *O. minutus*; 370 bp in *O. tantillus*; 356 bp in *O. nagaii*; 405 bp in *O. sauteri*; 424 bp in *O. majusculus*; 322 bp in *O. tristicolor*; 420 bp in *O. strigicollis*; 345 bp in *O. insidiosus*; 299 bp in *O. niger*, and 438 bp in *O. laevigatus*.

The sequence of ITS1 for the different *Orius* species was not able to be aligned because of the very high mutations this spacer exhibited. Meanwhile, the flanking gene of 18S rDNA was conserved within the genus. Because of this ambiguity, the relationship among the *Orius* species could not be constructed with clear resolution. In spite of the high ambiguity in the sequence of ITS1, nearly complete identity of this spacer between *O. albidipennis* and *Orius* sp-Taif strain was found. This identity supports that Taif strain clustered to the Egyptian *O. albidipennis* in spite of length difference and mutations (deletion and substitution) this spacer exhibited between the two taxa. Therefore, we recommend sequencing ITS1 and other mitochondrial DNA markers (cytochrome b gene) for more samples of both taxa in a further study in order to identify the unknown species of Taif and to fix its relation to the Egyptian species.

**Figure 3.**
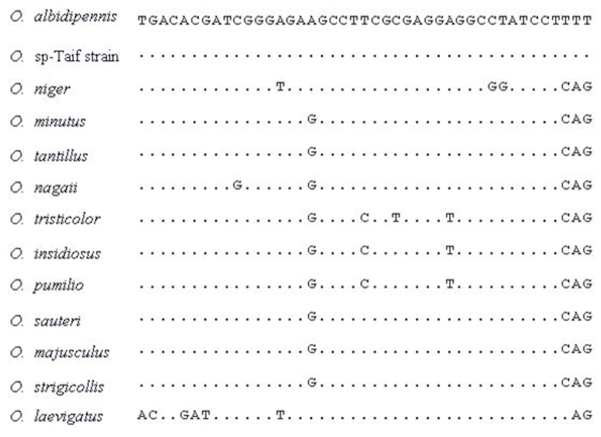
The alignable fragment of ITSI for different *Orius* species that was used in the analysis. High quality figures are available online.

**Figure 4.**
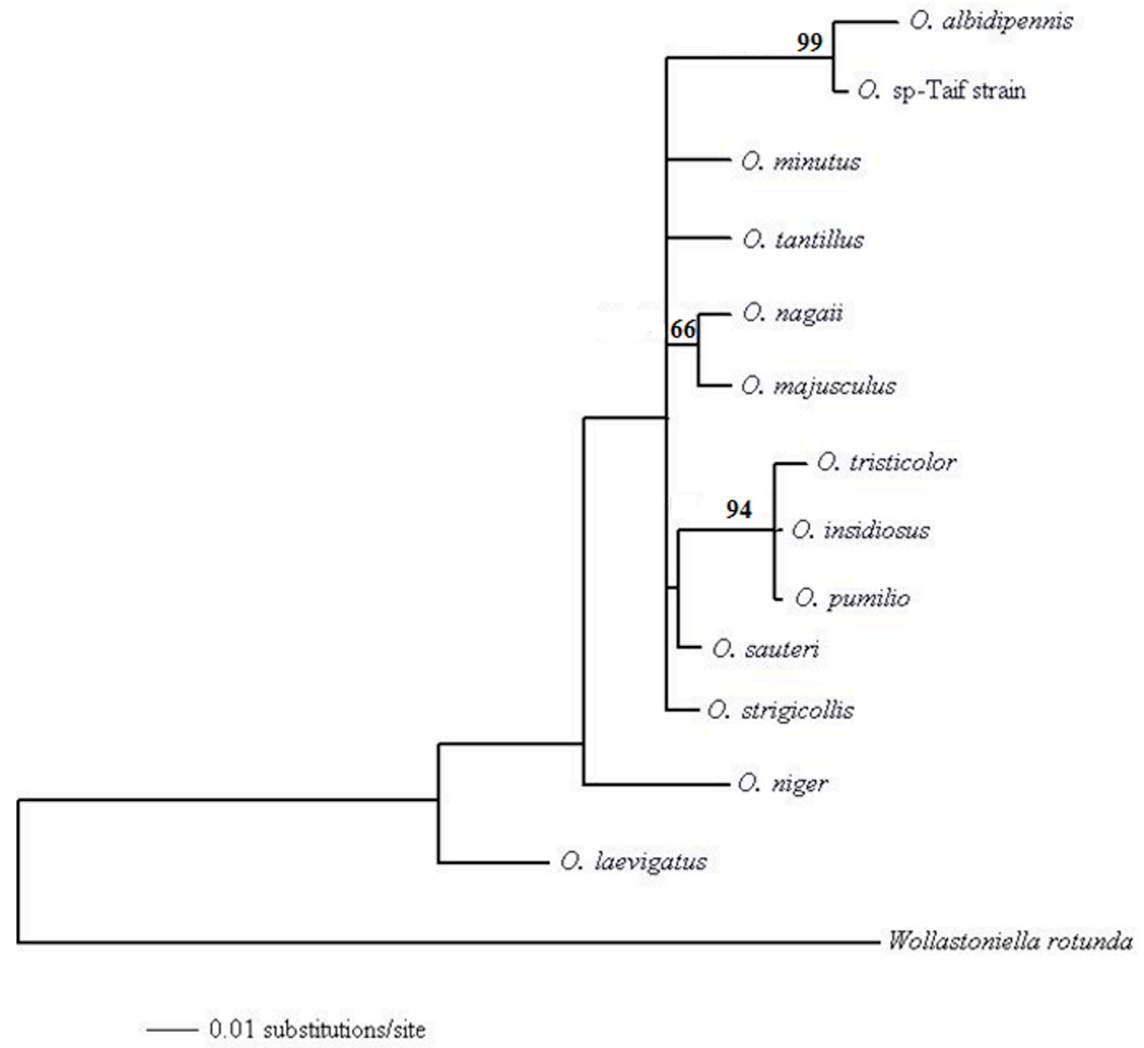
A maximum-likelihood tree for *Orius* species based on the alignable 18S and ITS1 sequences from various species. The bootstrap probabilities are shown at the nodes of interest. High quality figures are available online.

Analyses of ITS1 for different *Orius* species were conducted to assess their relative relationships after resolving the alignment problems for ITS1 of anthocorids (Honda et al. 1998) with the sequence identified here. 131 bp from 18S rRNA gene were concatenated with the alignable 43 bp from ITS1 (Figure 3) and were used for analysis. Therefore, unambiguous 174 nucleotides were used in analysis by maximum-likelihood method. Fifty-six substitution models were tried by using the Modeltest (Posada and Crandall 1998), and the best fit model that explained the data was GTR+I. A single maximum-likelihood tree was found with a negative log likelihood score —lnL = 430.6143 (Figure 4). The base frequencies were A = 26.5%, C = 21.8%, G = 31.0% and T = 20.7%. In the constructed tree, the *Orius* species found in Taif was segregated with the Egyptian *O. albidipennis* (99% bootstrap).

The Japanese *Orius* species (*O. minutus, O. sauteri*, and *O. strigicollis*) were grouped together as previously shown (Honda et al. 1998). In general, the relationship among different *Orius* species agreed with the previous constructed relationship (Honda et al. 1998; Jung et al. 2010; Shapiro et al. 2010) with differences in the positions of some taxa in our tree. These differences could be because only the manually alignable fragment of ITS1 was used in the present study, whereas the previous studies used more data from ITS1 and aligned it automatically by Clustal W program.

In conclusion, *Orius* sp.-Taif strain is closely related to the Egyptian *Orius albidipennis*, and both taxa could be from the same species. This result should be confirmed with sequencing more molecular markers for more samples of both taxa.

## Abbreviations

**ITSI**,: internal transcribed spacer 1
**PCR**,: polymerase chain reaction
